# The Institutional Library

**Published:** 1914-04-04

**Authors:** 


### THE INSTITUTIONAL LIBRARY.

### School Design and School Treatment.

Problems of School Hygiene: A Report of the First Conference of Scottish School Medical Officers. Edited by W. Leslie MacKenzie, M.A., M.D., LL.D., and Lewis D. Cruickshank, M.D., D.P.H. (Edinburgh and London: William Hodge and Co. 1914. Price 2s. 6d.)

In May last year the first conference of school medical officers ever called in Scotland was held in Edinburgh at the instance of the Scotch Education Department. Some seventy delegates assembled, representing every part of Scotland; these were not confined solely to school medical officers, but included the medical lecturers at the provincial training centres and several school inspectors of the Scotch Education Department. As a result the conference demonstrated the value of bringing the central department into touch with the local officers, and made manifest the idea that the whole education medical service is a unity. From the hygienic training of the teachers to the inspection of the children there is a continuity of work and method. A foremost place in the programme was given to the medical aspects of physical education, and the actual demonstration at the Dunfermline College of Hygiene and Physical Training was undoubtedly a fine feature of the conference. A thoughtful and well-balanced paper was contributed by Dr. Lewis D. Cruickshank on the subject of physical education. The author, who is the principal of the Dunfermline College, is a well-known authority on school clinics, and his highly instructive book on school clinics in general at present holds the field as an authoritative discussion of the function and organisation of these institutions. The book has been reviewed in our columns (November 1, 1913). The Chairman of the Dunfermline Carnegie Trust (John Ross, Esq., LL.D.) briefly outlined the evolution of the physical training work of the Trustees, which has culminated in the creation of the College whence qualified teachers pass on the "Dunfermline mark" to the children in the public schools throughout the country.

Of the papers read at the conference perhaps the most practical was that by Dr. A. Campbell Munro, Medical Officer of Health for Renfrewshire. He made some very pertinent criticisms on the generally accepted type of school with a central hall, and proffered a plan of a school embodying his own suggestions as to floor space, ventilation, heating, and construction. Dr. Munro's plans have the very important recommendation of economy; the cost per school place in the case of three pavilion schools built was £10 10s. 4d., as compared with £15, the mean cost of the central-hall schools previously built. Medical inspectors of school children who are suffering from the self-imposed monotony of the work will do well to read Dr. George Rose's paper on school medical research. They will find therein suggestions which ought to rouse them to an appreciation of the rich harvest which lies at hand, and which show how much can be done even by the efforts of a single individual. The volume also contains the interesting paper of Dr. Wm. Darling on the medical examination of candidates for the teaching profession, in which the difficulties of prognosis especially are discussed in relation to the regulations. Doubtless, as time goes on and medical knowledge in its various departments becomes better correlated, examiners will be in a stronger position in regard to passing candidates with such defects as a "mitral murmur." At present it is highly probable that too much is being made of such defects existing, as they so often do, in subjects apparently in the best of health. In such cases the extended use of the electro-cardiograph will in future play a reassuring part. The concluding papers, on the medical treatment of school children, disclose the fact that there exist in Scotland the same difficulties in obtaining adequate and co-ordinated relief as exist in England. This report of a conference which was productive of great mutual assistance to those concerned deserves attention from English education authorities, and such a meeting convened by the corresponding Board of Education in England would, we feel sure, receive cordial support by our school medical officers, and be equally advantageous in promoting throughout the whole service a spirit of good understanding and friendly co-operation.

Hæmocytes and Hæmic Infections: A Handbook for Students and Practitioners. By F. W. E. Burnham, M.D., C.M., Winnipeg. (London: H. K. Lewis. 1913. Price 25s.)

This pretentious volume may be regarded as an atlas of microphotographs of blood cells and blood parasites, rather than as an authoritative text-book on hæmatology. Not that the letterpress is lacking in readable qualities or in competent instruction, but that the very numerous photographic reproductions of highly magnified blood cells, etc., claim the chief attention of the reader. These microphotographs account for more than half the bulk of the book, and vary considerably in usefulness. Some are decidedly superfluous. Most are reproduced with an exceptionally large magnification—namely, 3,000 diameters—a feature of which the advantages are not always apparent. While many of the plates are mere duplications without obvious excuse, there are a few omissions which might profitably have been included; for example, a plate showing the granules found in the erythrocytes in cases of lead-poisoning. The photographs showing parasites are perhaps the most instructive; some of these are successful examples of a difficult art and reflect credit on the author, who is evidently an enthusiast in microphotography. The series comprises normal and abnormal blood cells, protozoa, bacteria, and filaria.

### The Problem of Mental Patients.

Mental Diseases. By R. H. Cole, M.D., M.R.C.P., Physician for Mental Diseases at St. Mary's Hospital, Lecturer on Mental Diseases at St. Mary's Hospital Medical School. (University of London Press. 1913. Pp. 343. Price 10s. 6d. net.)

The main requirements in a text-book on this subject for an ordinary medical student are that it shall be readable, clear, and concise; shall give him information that he will want for examination purposes; and that it shall not dip deeply into those philosophical and psychological problems which are only of interest to those who are going in for higher examinations on the subject. The practitioner, on the other hand, requires a text-book to help him to solve difficulties which occur in his practice, such difficulties as diagnosis, prognosis, and treatment, and also to give him advice on technical points of procedure in dealing with patients. To both students and practitioners, therefore, the discussion of deep philosophical and psychological problems can but cause trouble, since reading them seems a waste of time, and missing them out always leaves a sense of uncertainty lest some required information has been missed. The present volume appears to fulfil the requirements of both the student and the practitioner by the information it contains and the absence of the abstruse. The volume opens with a short chapter on the history of insanity, its recognition and treatment up to the present time. Then come chapters on psychology, diagnosis, ætiology, and classification. The succeeding chapters deal with the varieties of insanity and the conditions associated therewith, its pathology, prognosis, legal relations, and finally one on general treatment. This arrangement appears consecutive and good, nor does any individual chapter appear to be unduly drawn out. Psychology and its problems are treated in four chapters, but only to the extent that is required for a proper understanding of the descriptions of, and the differences between, the forms and varieties of mental disorders; and though the student may find that these chapters require very close attention, yet a careful study of them will repay him later. The classification of mental diseases used is simple.

The remaining chapters are in harmony with the earlier ones. Those important subjects, hysteria, neurasthenia, and psychasthenia, might perhaps have been treated more fully for the sake of the practitioner, for it is patients suffering from these conditions who so often cause him anxiety, and, unless tactfully handled, drift away from his hands into those of the quack. For the proper management of these cases a clear conception of the underlying mental condition is essential. The chapter on the legal relations of insanity and mental deficiency is brought up to date by including a description of the methods to be employed for dealing with mentally deficients under the new 1913 Act, which came into force on April 1. This is a most useful chapter, both as regards the information concerning certification for care and treatment and also for the paragraphs on testamentary capacity, civil liability, and criminal responsibility. There are, too, some useful hints about examining patients on these points. The last chapter, on care and treatment, with information as to single care, the different kinds of institutions, and the selection of patients for each of these, is one which will be useful as a reference. There are many diagrams, charts, pictures, and plates of pathological specimens, which are well chosen and reproduced. The indexing is good, the print clear, and misprints conspicuous by their absence, only one being noticed (p. 15). The most unfortunate feature of the book is its weight.

### The Prevention of Deafness.

The Prevention of Deafness. By Adair Dighton. (London: The British and Foreign Deaf Association, 31 John Street, Bedford Row, W.C.)

It is even to-day a lamentable fact that it is not sufficiently realised that deafness and ear disease lie just as much within the realm of preventive medicine as the control of scarlet fever or tuberculosis. Notwithstanding the progress of aural surgery in modern times, which has doubtless been the means of saving many lives, preventive otology has even a greater field of usefulness. The British and Foreign Deaf Association of 31 John Street, Bedford Row, W.C., has just issued a popularly worded pamphlet on the prevention of deafness, written by Dr. Adair Dighton. It is hoped, as there is no charge for this brochure, that its dissemination by school doctors and school nurses will do good, if only in its warning against domestic tinkering with ear troubles, including the frequently occurring "foreign body." The danger of sea-bathing and diving without first protecting the ears is insisted upon. It would, however, have been well if Dr. Dighton had been more explicit in giving practical directions as to how the ears should be protected, for unless this is done properly it is futile, and may even be harmful.

Carbon Dioxide Snow. By J. Hall-Edwards, L.R.C.P., Hon. F.R.P.S. (London: Simpkin, Marshall, Hamilton, Kent and Co. 1913. Pp. 78. Price 3s. 6d.)

Carbon Dioxide Snow has now been before the public for some years as an agent for curing many superficial skin lesions, and its powers and properties are quite familiar to the experts. A special monograph is therefore a necessity, and, as a matter of fact, Dr. Hall-Edwards has been forestalled some years ago by Dr. Cranston Low; so practitioners have now two books on the subject to choose from. For clearness, brevity, and general breadth of view we can highly commend this book. General practitioners in large towns, however, will find they can get their snow as required from surgical-instrument makers, without any necessity for purchasing the rather elaborate outfits for making it at home; the latter are all very well for specialists who are constantly needing the snow, but are unnecessary for the doctor who uses it once or twice a year.

### Two Volumes on Midwifery.

Notes on Midwifery. By C. F. Lassalle, M.D. (Edinburgh: W. Bryce. Pp. 126. Price 2s. 6d. net.)

The author of this book practises in the West Indies, and the printed notes of his lectures to midwives there have attained such local popularity that they are now being offered to a wider audience. As far as they go—the teaching is necessarily elementary—the notes offer an excellent synopsis of those parts of the science of obstetrics which a midwife ought to know. The publisher has done his share, too, by a particularly good arrangement of type. A glossary of obstetrical terms at the end is also a useful feature of the book.

A Synopsis of Midwifery. By A. W. Bourne, M.B., F.R.S. (Bristol: John Wright and Co. 1913. Pp. 212. Price 5s. net.)

As a help to students in a last revision of the subject of midwifery before sitting for an examination in that subject, this synopsis will certainly prove useful, for it is well arranged and trustworthy. But we do not think the author is right in supposing that it will also be of value to the practitioner, who does not need the academical information of the earlier chapters, and ought to know by heart the later chapters on practical treatment. However that may be, the author is entitled to credit for a condensation of the science of midwifery, which is sound in essentials and avoids extreme views. Here and there, necessarily almost, individual teachers may not agree with the methods suggested; but the dogmatic note of a student's class-book precludes much discussion of controverted topics. We should have been glad to see the author adopt the method of reckoning all intervals of the gestation period in lunar rather than calendar months; and we demur to the bare statement that quickening occurs about the sixteenth week. Nor do we concur in the recommendation to restrict carbohydrates and fluids in the dietary of pregnant women with the object of reducing the size of the fœtus. But a few minor points of criticism such as these do not affect our verdict on the synopsis in general, which is decidedly favourable.

Compendium of the Pharmacopœias and Formularies (Official and Unofficial). By C. J. S. Thompson. Fourth Edition. (London: J. Bale, Sons and Danielsson, Ltd. 1913. Price 5s. net.)

This reference book is intended for pharmacists, students, and medical practitioners. In form it has some resemblance to another and very well-known pocket manual. It contains a very large amount of pharmaceutical information, including synopses of the French, German, American, and British Pharmacopœias. There are also given the usual tables of dilutions, doses, metric measures, gravities, solubilities, and the innumerable odds and ends that the pharmacist is supposed to expect in a book of this kind. On the subject of wine analysis the author has not been very successful with his condensed paragraphs; the methods described would not be free from fallacies. Some brief remarks of little utility on the length of isolation usual in certain infectious diseases are erroneously referred to as quarantine periods. A lengthy list of popular synonyms for certain drugs and preparations may be considered a useful feature, but the list of Latin terms used in prescriptions contains the usual number of quite unnecessary expressions.

### A History of Ambulance Work.

The Errand of Mercy. By M. Mostyn Bird. (London: Hutchinson & Co. 1913. Pp. 348. Price 3s. 6d.)

This volume endeavours, not without success, to present a synopsis of the care of the wounded in warfare from the earliest times down to the present day. No originality is claimed for it, and it is a frank compilation from many sources, which are duly acknowledged. It is an elementary text-book of its subject, suitable for general readers who are interested in ambulance work of all kinds. When the author is propounding individual views and opinions, instead of detaching cold facts derived from authentic sources, there is a strong tendency to exaggeration and sentimentality and even to palpable misstatement. The illustrations are exceedingly poor, and should have been omitted. Otherwise the book is quite good within its limitations.

Trachoma and its Complications in Egypt. By A. F. MacCallan, M.D. Camb., F.R.C.S. England. (Cambridge: University Press. 1913.)

The title of this book will attract a less number of readers than its contents merit. We have been accustomed to regard trachoma as a disease in which we have little interest unless our work leads us into the regions in which it has become historic. Perhaps many of us required the reminder that the older members of our profession had chronicled its spread to Greece and Rome, and that the establishment of our own Ophthalmic Hospital at Moorfields in 1804 was due, in large measure, to the spread of the disease among our own soldiers on their return from the Napoleonic campaign in Egypt a few years previously. The author gathers interest round his demonstration of the liability of the Egyptian stamped upon him by centuries of inheritance. He admits that in many cases no bacteria can be found in the initial stage—the mark of a weakened defence; but he proceeds to demonstrate that commonly found forms of microscopic life are needful to complete the picture of trachoma as it exists. To the gonococcus he assigns a very large importance, but he also indicates the influence of the Klebs-Löffler bacillus. The Koch-Weeks and Morax-Axenfeld microbes will interest British readers less than the prominence given to overcrowding and dust as predisposing factors. The latter conditions exist in the crowded areas in London, and especially among the cloth workers in the Jewish quarter, where the disease is far from unknown. There is no prolixity in Dr. MacCallan's writing; each page increases in interest, and holds the reader to his task, while the full index keeps a large subject within the reach of ready reference.

### Babyhood Literature.

Our Baby. By Mrs. Langton Hewer. (Bristol: J. Wright and Sons. 14th Edition. 1913. Pp. 192. Price 1s. 6d. net; leather, 2s. 6d.)

About Baby. By Francis Tweddell, M.D., and W. Barkley, M.B. (London: Mills and Boon. Pp. 148. Price 1s. net.)

The first of this couple is a very old friend; the other an American book which is now being published in an English edition. Mrs. Hewer's excellent volume has been appreciatively reviewed more than once in these columns; and it must be very gratifying to her to find each recurring edition so rapidly exhausted. We note that this edition completes the hundredth thousand, and that much new material has been incorporated since the last one, especially on the subjects of tuberculosis and of baby management in the tropics. This latter chapter is, we think, rather a mistake; it is impossible to sum up within ten pages the subject of tropical hygiene and tropical medicine as applied to babies, and it would have been wiser not to attempt it. For instance, limits of space (presumably) have led the authoress to make the brief statement that the parasite of malaria can be found under the microscope in a drop of blood taken from the finger. This statement is, in itself, incomplete and inexact; and, even if it were not, we cannot see what value it can have in a book addressed to young mothers and nurses. Nor, again, is it an "old saying" that where there are no mosquitoes there is no malaria. When Mrs. Hewer's book was first published no such saying was current, though everyone knows it now.

Dr. Tweddell's book, we are told by his editor, has been abbreviated by the omission of certain matter which is considered unnecessary in view of the conditions prevailing in Great Britain. It is a clearly written, intelligible, straightforward summary of the subject, which can be placed in the hands of mothers and nurses without any misgivings. For our own part, we think most of the voluminous babyhood literature errs by entering much too fully into children's diseases—the infectious fevers and the like. In this respect we find little cause of complaint against Dr. Tweddell, though we should certainly have omitted the paragraphs on scabies if we had been exercising Dr. Barkley's editorial function. "Hives" as a substitute for "nettle-rash" is surely an Americanism which might also have been expurgated. The only other piece of advice which seems open to criticism is that to circumcise children up to four months of age without an anæsthetic.

Rearing an Imperial Race. Edited by Charles E. Hecht, M.A. (London: Published for the National Food Reform Association by the St. Catherine Press.)

This book consists of a compendium of information relating to the educational aspect of food and diet. It does not pretend to be an original contribution to the literature on the subject, but reproduces a great variety of information, official and otherwise, bearing on the subject. It should prove of value to school medical officers and others concerned with the administration of this side of educational work. The proceedings of the second Guildhall Conference, held last summer under the auspices of the National Food Reform Association, are reproduced in full, and form a valuable record of the opinions and experience there related by many of the leading spirits in this field of public work.

